# “Opening a Door to a New Life”: The Role of Forgiveness in Healing From Moral Injury

**DOI:** 10.3389/fpsyt.2018.00498

**Published:** 2018-10-16

**Authors:** Natalie Purcell, Brandon J. Griffin, Kristine Burkman, Shira Maguen

**Affiliations:** ^1^San Francisco Veterans Affairs Health Care System, San Francisco, CA, United States; ^2^Department of Social and Behavioral Sciences, University of California, San Francisco, San Francisco, CA, United States; ^3^Department of Psychiatry, University of California, San Francisco, San Francisco, CA, United States

**Keywords:** moral injury, military veterans, forgiveness, self-forgiveness, psychotherapy

## Abstract

For military veterans struggling with moral injury, forgiveness can become both an animating concern and a potential path to healing. In this perspective piece, we draw on our clinical work and research findings to examine why forgiveness matters to veterans who feel guilt and shame about their actions in war, what type of forgiveness is attainable and meaningful, and what role clinicians can play in facilitating forgiveness. We conclude by reflecting on the potential, as well as the limits and tensions, of forgiveness work in the context of military moral injury.

“[Moral injury] is the raw primitive feeling I did something terribly wrong and I just don't know whether I was justified or whether I can be forgiven. The cure has to involve the honesty to acknowledge, yes, I did this.”—Father Thomas Keating, *Almost Sunrise* [quoted in (1)]

If healing from moral injury begins with an honest acknowledgment of one's actions, how can mental health professionals support the multi-faceted healing process to follow? We argue that forgiveness—especially self-forgiveness—is the cornerstone of this process, helping veterans to work through their guilt and shame, honor their violated values, re-engage with family and community, and gradually restore an integrated moral identity. In this perspective piece, we describe why forgiveness is both difficult and crucial for military veterans who feel guilt and shame about their actions in war. We explore what type of forgiveness is attainable and meaningful, how the forgiveness process unfolds, and what role clinicians can play in facilitating forgiveness. We conclude with brief reflections on the potential and limitations of forgiveness work in addressing military moral injury.

Throughout, we reference the Impact of Killing (IOK) treatment program (2), a 10-week psychotherapy intervention developed by San Francisco VA clinicians and researchers to help military veterans struggling with moral injury after killing in war. We base our recommendations on our clinical experience with morally injured veterans, our prior research on military moral injury, and the voices of veterans who participated in our mixed-methods IOK studies (3–6).

## What does forgiveness have to do with moral injury?

“All I knew is I hurt inside and I didn't know why… I didn't know why I should feel so bad if I didn't do anything wrong. And then struggling with, well, did I do something wrong?”—combat veteran, IOK study

Wounds to the spirit or soul^1^ can be among the most devastating and enduring wounds of war. In recent decades, scholars have worked to characterize these moral wounds and to describe their impact on the lives of military veterans (10–12). From their work, we have learned that veterans can be ashamed, alienated, and disillusioned after returning from war, sometimes questioning their worth and goodness as human beings. Combat veterans can feel like war awakened their “dark side”—a “beast” or a “monster” that remains within, belying any sense of the self as a good person, a kind spouse or parent, a gentle and caring friend (5). Some morally injured veterans engage in years, even decades, of self-punishing behavior, often with only a vague sense of what is driving it. Some sabotage their relationships, employment, or other sources of potential happiness, feeling that they don't deserve anything positive or fulfilling in life. They may find themselves emotionally numb or racked with anger or despair that has no clear cause or target. Those with the most serious moral wounds isolate themselves from intimate relationships and avoid people and things that once had meaning for them, sometimes losing themselves in the haze of drugs, alcohol, or prescription medications (5, 13, 14). Some consider ending their own lives, and some ultimately do so (15).

The central premise of this paper is that recovering from the most serious moral wounds of war entails seeking and receiving forgiveness—particularly self-forgiveness. In the context of military moral injury, this is a complex and controversial claim. After all, to suggest that healing from moral injury entails forgiveness is to imply that there is some wrong to forgive, and this is often ambiguous. In combat, violent actions that are considered immoral in most other contexts become, instead, one's duty. These actions become, also, the basis for protecting oneself and one's fellow soldiers^2^ from grave harm. Often, warriors must make split-second decisions—for instance, to shoot or not to shoot—with life-or-death consequences. Those decisions are often fraught with moral complexity and are made under intense pressure. In these contexts, right-and-wrong is by no means black-and-white.

War entails lethal violence, but moral principles like public service and personal responsibility, as well as civic ideals like freedom and democracy, can underlie the choice to serve for many young men and women. When they do serve, values of loyalty, compassion, and camaraderie often motivate their actions, especially when they act to defend the lives of their fellow soldiers. In war, these affirmative moral values can become reasons to do violence, to kill, or to take other actions that would be considered serious moral violations in civilian life. Yet, the very same values can also create compassion for the human beings serving in the opposing army and for the civilians whose lives are affected by war. The very same values can cause soldiers to wonder whether they did or did not have a choice when they followed troubling orders. The very same values can cause some to question the underlying mission of the war they are fighting—are they really serving freedom, democracy, and justice?

Questions like these may not arise until long after the battle, or even the war, is over. Like all humans, soldiers are not only moral creatures; they are also embodied beings, whose actions may be shaped by fear and adrenaline as much as conscious thought. In the heat of combat, a soldier may make the choice to shoot or kill, realizing only afterward that he did so prematurely and an innocent person died as a result. Or an officer may issue orders that she once believed would serve a greater good, but later finds herself doubting whether the ends justified the means. Many of the men and women who go to war are young adults thrust into an environment that is literally and figuratively foreign. They may find themselves facing serious threats to life and limb, watching their comrades face the same, and bound to follow orders or face punishment, disgrace, and ostracism. If they volunteered for duty, they may find themselves in this situation by virtue of their own choices—a layered moral universe where the matter of responsibility is not easily settled.

Given the moral complexities of war and the pressures that soldiers face when serving their country, can they be deemed morally responsible for actions they took or failed to take in war? Can be they be considered guilty of any moral wrong that needs to be forgiven? In many cases, we have found, only the soldier can answer these questions, and only after a sincere and thoughtful reckoning with the moral questions deferred in the heat of combat. For some, that reckoning results in a cognitive reappraisal that, in itself, eases guilt, shame, and suffering, revealing that there really is no deep moral failing to forgive. But, for others, there remains a debt to settle, and the price of that debt may be the enduring guilt and shame of moral injury.

War does, after all, entail moral choices. Those choices may be made under extraordinary constraints and pressures, but they are made by individuals with varying degrees of agency and freedom. An evaluation of one's actions in war may indeed lead to the considered and thoughtful conclusion that a wrong was committed. Sometimes, those wrongs are serious and unequivocal; other times, more subtle and nuanced. But no context, even war, provides blanket absolution for human actions and their consequences.

We argue that, when the wounds that one suffers from are indeed moral wounds—when the guilt and shame consuming one's conscience stem from actions that one took or failed to take in war—the healing process must involve moral growth and reconciliation. Here, we speak of a reconciliation between the values one wants to hold and the actions one has taken; between the person one wants to be and the person one has been; between the ethos of a soldier at war and that of a veteran who has returned home. As Father Keating suggests, the process of reconciliation begins only when one looks at his own actions with eyes wide open (1). If he assesses that he did in fact make choices or take actions that are not compatible with the person he wants to be, we argue that he must seek and find forgiveness before he can heal.

## What sort of forgiveness is attainable and meaningful?

“I hated myself for what I did and all these years I've taken that hatred with me.”—combat veteran, IOK study

We speak of forgiveness as a process of emotional growth, release, and transformation that can facilitate reconciliation in the aftermath of a significant moral violation. It is an active, morally-engaged process that requires both acceptance *and* change. As Webb et al. (16) have written, “Forgiveness occurs over time and is a deliberate, volitional process involving a fundamental shift in affect, cognition, and/or behavior;” this shift entails releasing “ill will… without condoning, excusing, or denying the transgression(s)” (p. 220).

Whether forgiveness is needed and, also, what sort of forgiveness is necessary and meaningful, is a deeply personal matter and one that often requires painful exploration of the consequences of one's actions and the harm done to others. Sometimes, a veteran will feel that he needs the forgiveness of those he harmed or killed in combat; sometimes, the forgiveness of his God or a higher power; sometimes, the forgiveness of loved ones he has alienated after returning home. But the veteran is likely to find no clear subject who is positioned to forgive the combat actions at the heart of his moral injury. After all, who can and should offer forgiveness for wrongs committed against anonymous others half a world away—others who may be alive or dead?

We contend that, with whomever else a veteran feels he must reconcile, the heart of healing from moral injury is a process of forgiving the *self* —that is, of reaching an inner reconciliation where one acknowledges and attempts to makes amends for any harm done, while also recognizing the self as a fallible person engaged in continuing moral growth and development. For Cornish and Wade (17):

“[S]elf-forgiveness [is] a process in which a person (a) accepts *responsibility* for having harmed another; (b) expresses *remorse* while reducing shame; (c) engages in *restoration* through reparative behaviors and a recommitment to values; and (d) thus achieves a *renewal* of self-respect, self-compassion, and self-acceptance” (p. 97).

Here, self-forgiveness is definitively not about excusing one's actions, explaining them away, or simply forgetting them and moving on. That would constitute an inauthentic forgiveness that is not compatible with healing from true moral wounds and can, instead, compound or prolong moral injury. Authentic or genuine self-forgiveness, by contrast, is an often-painful process that entails a moral reckoning as the precondition for spiritual growth and renewal (18, 19).

The word “process” is central to our understanding of self-forgiveness. It is not an act or a gesture, but an emotional and behavioral regeneration that requires moral engagement and change (20). One veteran in IOK treatment compared the process to unpacking a rucksack that he had carried on his back in combat—removing and examining its weighty components one at time, gradually unburdening himself and making it possible for him to *move* and to *act* differently—in his case, to better connect with and care for neglected others in his life, even if the sack would always remain on his shoulders (2). As Webb and colleagues (16) have written:

“Self-forgiveness occurs over time and is a deliberate, volitional process initiated in response to one's own negative feelings in the context of a personally acknowledged self-instigated wrong, that results in ready accountability for said wrong and a fundamental, constructive shift in one's relationship to, reconciliation with, and acceptance of the self through human connectedness and commitment to change” (p. 221).

Authentic self-forgiveness is not a linear process, but one filled with ebbs and flows. Sometimes, what one takes out of the rucksack goes back in for a time. And nothing removed is ever forgotten.

## How does self-forgiveness begin?

“I felt like a monster. I felt like a monster separated from the human race”—combat veteran, IOK study

Moral guilt is often conceptualized as a *constructive* negative emotion—one that can catalyze behavioral change and lead to personal growth. Guilt is, in fact, an important precursor to the transformational experience of authentic self-forgiveness (21). Yet, when guilt becomes an enduring, global criticism of one's self and one's behavior—when guilt becomes indistinguishable from chronic shame—it is no longer associated with affirmative change, amends-making, or personal growth. It can become, instead, a source of moral paralysis and other psychological and behavioral problems, including the self-punishing behaviors associated with moral injury (18, 22). This is often the case among morally injured veterans, who can become locked in patterns of self-hatred, self-condemnation, and self-punishment, perceiving no way out (5, 23).

The source of this moral quicksand, we argue, is an inability to see any path toward the reconstitution of a self-worthy of respect and love—a morally intact self. When a veteran has committed, in his or her eyes, a wrong so significant that it defines the moral self and cannot be corrected, it may seem like there is no viable path forward. At this juncture, to broach the topic of *self-*forgiveness is to introduce the possibility that such a path can be forged *and* to suggest that the guilty party must take the first active steps to forge it. For the morally injured and ashamed veteran to take those steps, he or she must first understand the meaning of authentic self-forgiveness and have some sense of the process that it entails. The veteran must also recognize self-forgiveness as distinct from the morally stagnant practices of excusing or condoning one's actions—practices already rejected by those who are sincerely remorseful.

There are extraordinary barriers to reaching even this modest starting point. Those barriers may include the veteran's sincere convictions that some acts are unforgivable, that only victims can forgive, or that forgiving is tantamount to letting oneself off the proverbial hook. Ideas about self-forgiveness are often embedded in one's cultural, spiritual, or familial background, and some veterans may resist the concept itself, believing that self-forgiveness is meaningless or self-indulgent, or perhaps that forgiveness can come only from a higher power. Even those who embrace the concept may confront other barriers, including obstacles to making direct amends to those harmed by their actions in combat. Ironically, the veteran's own recognition and articulation of these barriers can provide evidence of an intact moral self that belies the image of the self as an irredeemable moral failure. Articulation of barriers to self-forgiveness also empowers the veteran to begin analyzing and disentangling destructive beliefs about the self, finding small openings that illuminate a potential pathway to the restoration of moral identity and self-regard—a pathway that must ultimately honor the veteran's most deeply held convictions and values.

In our work, we have found that the core components of the self-forgiveness process—accepting responsibility, cultivating self-compassion, making amends, and reconstructing an intact moral identity—are near-universal steps on the pathway through and beyond moral guilt. For most veterans, recognition and reaffirmation of violated values, such as respect for the sanctity and dignity of human life, are essential to the process. So too is reparative work to make right what was wrong. Because veterans are seldom able to make amends directly to those harmed or killed in war, they may find ways to affirm their values through service to the broader community—for example, joining organizations to help other veterans of war, performing community service or volunteer work as part of a religious congregation, speaking in public or to groups of school children about their experiences and lessons learned, or even raising their own children to respect the values they feel they violated during their service. Some veterans even return to the site of their most traumatic experiences—for instance, traveling back to Vietnam to pay respects to the dead and to atone for their actions in war. Taking steps like these can help veterans begin to move from a place of shame and guilt to one of self-compassion, moral renewal, and hope.

A marker of whether the self-forgiveness process has started is observable changes in functioning: is the veteran able to have better relationships, to reconnect with their spiritual community, to speak about topics they considered unspeakable in the past; to visit places they have been avoiding? In our work with morally injured veterans, we look for these signs of progress but also recognize that self-forgiveness is an ongoing process that will continue after any formal treatment program ends. In particular, healing must continue across the contexts that are most meaningful for the individual, including within their personal relationships, families, and communities. For this familial and social reintegration to take place, there must first be meaningful progress toward self-reintegration—the gradual reconstitution of a coherent moral identity on the path toward self-forgiveness.

## How can self-forgiveness help?

“I feel like I have let go… like I don't have to be in Vietnam again. I'm in a present state right now.”—combat veteran, IOK study

We believe that embarking on a journey of authentic self-forgiveness unlocks the possibility of re-engagement in one's life and one's community after moral injury. Recent studies show that the IOK treatment program (2), which centers on self-forgiveness, can help morally injured veterans feel less depression, anxiety, suicidality, and shame (3, 4). After completing the self-forgiveness modules of the IOK program, veterans often described feeling a sense of profound relief. “It's freedom from being captive,” explained one veteran, “It's not that I am guilt free or shame free; it's just that I am not packing around all that load, that weight… How do you describe opening a door to a new life?” Some described being able to open up emotionally and become intimate with loved ones again, and others spoke of feeling less anger and more compassion toward others. Many affirmed that self-forgiveness was the heart of their healing process, enabling them to love others and to find compassion for themselves.

Emerging scholarship supports our contention that self-forgiveness has the power to change the lives of morally injury veterans. Although there is little research on the impact of *receiving* forgiveness, to forgive is clearly associated with psychological wellbeing, including less depression, anxiety, and shame (24). Research also suggests that *self*-forgiveness is associated with lower levels of anxiety, depression (25), and suicidality (26), fewer destructive behaviors including problematic substance use (27), more satisfying and committed relationships (28), and other improvements in both psychological and physical health and wellbeing (24). In short, forgiving the self can also help heal the bodies and minds of morally injured veterans.

## What role can clinicians play in facilitating forgiveness?

“[I] have to look into your eyes and see that you really care.”—combat veteran, IOK study

Forgiveness is a complex process with psychological and spiritual dimensions, and some might consider it outside the purview of mental health clinicians. Indeed, it is more familiar territory for chaplains and clergy, who have supported veterans in finding forgiveness and healing from moral injuries long before clinicians began using the term “moral injury” (29). Nonetheless, we argue that mental health clinicians, especially those who work with veterans through VA or Department of Defense healthcare systems, can play a crucial role in facilitating the processes of self*-*forgiveness and reconciliation at the heart of healing from moral injury.

When working with morally injured veterans, the role of the clinician is first to create a space where veterans can begin to appraise the traumatic events at the foundation of their shame and guilt. To do this, it is essential to establish a trusting, nonjudgmental relationship and to convey that no topic is off limits for thoughtful and compassionate discussion. Invited to comment on their IOK treatment experience, veterans routinely emphasize how important the “therapist connection” is to them. As one veteran explained, moral injury work “can't be an intellectual exercise”: “Whatever it takes to have that safe good connection between veteran and therapist, that has to be there before you can go a useful distance into exploring forgiveness.” To open up about the sensitive topics of shame, guilt, and moral injury, the veteran must feel confident that her mental health provider can remain present, engaged, and compassionate, even when the discussion ventures into the most dark and graphic of subjects.

Creating a space for open, compassionate exploration requires resisting any personal judgments about the veteran's actions and appraisals, and ultimately honoring the veteran's own moral values and judgments. At the same time, the clinician should play an active role in encouraging self-exploration and ask critical questions about unexamined beliefs and assumptions. An engaged clinician will help the veteran examine personal beliefs about specific morally injurious experiences, encouraging attentiveness to context as well as consequences. Clinicians can also ask questions that encourage patients to think more flexibly and compassionately, helping them find a balance between acceptance and change. In a treatment context, this work of self-examination and reappraisal is the foundation for authentic self-forgiveness work, and it is often necessary before explicitly broaching the topic of forgiveness.

Ultimately, we have found that it is important to raise the matter of forgiveness directly. In the IOK model, we initiate this process by inviting discussion of the personal meaning, cultural relevance, and spiritual significance of forgiveness for each individual veteran. We also invite exploration of potential psychological and cultural barriers to self-forgiveness. We then work with the veteran to create a personalized, patient-driven “forgiveness plan” that is designed to transcend these barriers and to serve as a springboard to the self-forgiveness process. The plan is action-oriented and includes activities centered on examination and reaffirmation of values, such as written and verbal exercises inviting the veteran to define self-forgiveness, to delineate cultural beliefs about forgiveness, and to conceptualize how they have applied forgiveness to the self and others. Veterans in IOK treatment also develop an amends plan, identifying specific actions they can take to reaffirm their violated values and to live as the kind of person they want to be.

We have found that self-forgiveness work can be facilitated by incorporating selected tools and exercises of cognitive behavioral therapy into each veteran's forgiveness plan. Cornish and Wade (17) suggest encouraging patients to dialogue with parts of themselves and/or with others whom they've hurt, sometimes adopting or trying on different perspectives to encourage cognitive flexibility, empathy, and compassion. In IOK treatment, veterans are invited to write letters to those they have killed or harmed, letters to a younger version of themselves, and other letters tailored to highlight different perspectives and needs. Veterans report that these letters are often a catalyst for transformation, facilitating cognitive change, compassion, and awareness of the personal growth that has taken place since the war.

Throughout the treatment process, clinicians must be aware of their own values and judgments and be wary of any strong feelings that could disrupt the process. This is more easily said than done: veterans will sometimes express sentiments rooted in personal, cultural, and spiritual traditions that are unfamiliar, or even distressing, to the clinician. The veterans' values might also result in self-appraisals that the clinician feels are harsh or unwarranted. In expressing compassion, a clinician may be tempted to excuse or condone the veterans' actions—for example, by reassuring the veteran that their actions were justified. This form of reassurance is well-meaning but can hinder progress. We have seen veterans continue to harbor the same feelings of self-condemnation and shame, but simply avoid admitting them to a provider focused on reassurance. Clinicians should also avoid inadvertently steering veterans toward inauthentic self-forgiveness, which can delay real forgiveness work, create confusion between authentic and inauthentic self-forgiveness, and hinder eventual engagement in a more authentic process.

By facilitating initial progress toward self-forgiveness, the clinician can play a crucial role in helping veterans begin to heal from moral injury—a process that will continue long after treatment ends. As veterans pursue their forgiveness and amends plans and prepare to continue the work of self-forgiveness after treatment, part of the clinician's job is to make sure that each veteran has the necessary support in place and to help him or her build new support as needed—for instance, by encouraging the veteran to strengthen existing bonds with family and friends, or to forge new bonds within supportive veteran or spiritual communities. Clinicians can also facilitate veterans' connections to pastoral care through, for example, referrals to or collaborations with chaplains or clergy (e.g., moral injury groups that are co-led by mental health professionals and clergy). In these ways, clinicians can empower veterans to keep making progress on the path of self-exploration, community reintegration, and making amends.

## What are the limits of forgiveness?

“I can't forgive myself… I did something wrong”—combat veteran, IOK Study

It bears noting that not every veteran will feel that forgiveness is warranted or possible. Some will feel that their actions are unforgiveable. This may be especially true for veterans who killed civilians, participated in massacres, or took actions that can only be described as murder. Others may feel that they are not authorized to forgive their own immoral actions—believing, for example, that only victims can grant forgiveness. As one veteran in our IOK study said, “I can forgive people for what they've done against me, but I can't forgive myself for what I've done against somebody else.” These are serious moral concerns without easy resolution. Philosophers have long debated who has standing to forgive and whether any act is finally unforgiveable (30). However, when it comes to *self*-forgiveness, we have found that the individual veteran is ultimately the arbiter. A clinician may ask probing questions to encourage more critical and flexible thinking or greater attentiveness to context, but must finally respect the veteran's choice to embrace, or not to embrace, self-forgiveness as a goal. Some will choose to reject it.

Those who choose to pursue self-forgiveness are likely to find that it is a long journey with many ups and downs. That journey may result in worsening guilt and shame at first, and guilt is seldom resolved entirely, even in the aftermath of self-forgiveness. For many veterans, additional therapeutic work will be necessary to address the long-term traumatic impact of moral injury, which is often entangled with post-traumatic stress in complex ways. For others, religious or pastoral care may facilitate healing and spiritual growth beyond what clinical care can offer. Self-forgiveness work should not be conceptualized as the only approach to resolving the multiple psychological, emotional, behavioral, and spiritual problems that may be associated with moral injury.

There is also, as noted, some risk of inauthentic or “pseudo” self-forgiveness. If embraced uncritically or inauthentically, self-forgiveness can result in eased feelings of guilt that do not actually lead to reconciliation or amends, nevermind spiritual growth and learning (21, 31). Inauthentic self-forgiveness is also compatible with ongoing self-destructive behaviors, such as the self-sabotaging behaviors and substance abuse that can sometimes accompany moral injury (32).

Even authentic self-forgiveness has its limits. It can help some veterans reach a place of spiritual restoration, where they can live beyond shame and self-punishment. It can also help them re-engage with their families and communities and give back in meaningful ways that honor their values. But it can never undo what happened and is thus limited in its capacity to ease the pain of others who were harmed or victimized. This is particularly true when it comes to the moral violations of war, which often involve killing and harming anonymous strangers. In some sense, the most serious of wrongs can go un-righted, even in the wake of authentic self-forgiveness. Although the self-forgiveness process involves making amends and giving back, it is still primarily a matter of *personal* growth and transformation. In itself, it does not help to change the social or political conditions that lead soldiers into morally compromising positions and it may allow those conditions to continue unabated.

In the end, moral injury is not exclusively a psychological matter, and healing moral injuries requires more than the tools of psychology or psychiatry can offer. It requires spiritual growth rooted in both personal and communal values, as well as reintegration into a moral community (be it religious, secular, familial, or other). Often, there is an explicit social and political dimension to this healing process. For example, some veterans may feel that making amends entails seeking justice and contributing to specific communities in specific ways—a path akin to those created through restorative justice programs. Others might argue that the civilian community shares responsibility for the violence of war and, thus, that healing requires a *collective* reckoning with war's consequences (e.g., a truth and reconciliation commission). After all, soldiers suffer from moral injury as a result of actions they took in wars engineered by much larger political and social forces. For individual veterans, self-forgiveness is not a panacea, nor does it resolve the larger moral questions raised by the violence of war.

## Conclusion

“I had to learn to love myself. At one time I couldn't love myself… I had to forgive myself.”—combat veteran, IOK study

Forgiveness of the self is a powerful, if partial, intervention that can facilitate healing from moral injury. Although it does not constitute the totality of that healing process, we have found that it is a crucial springboard to the reaffirmation of violated values and the reconstitution of an integrated moral identity. Self-forgiveness may not repair the underlying conditions that leave so many soldiers affected by moral injury, but it can give individual veterans the opportunity to find a livable path forward. Clinicians, if they are willing and humble, can play a crucial role in facilitating the process of self-forgiveness. They can create a space for open and compassionate exploration of painful moral traumas, and help veterans chart a course toward the renewal of their moral self. Many veterans, we have found, can and do achieve that renewal—honoring their values, making amends to those they harmed, and finding ways to respect the self they have become.

## Ethics statement

This is not an original research manuscript, but it does reference findings and data across several of the authors' previously published studies. All studies described in this manuscript were approved by the UCSF Institutional Review Board, and all human subjects completed an IRB-approved informed consent process.

## Author contributions

NP, BG, KB, and SM all contributed to planning, development, and writing of this manuscript. SM and KB developed the clinical intervention (IOK) described herein. SM designed and served as principal investigator of the original research studies referenced throughout, with NP and KB conducting data analysis and interpretation. BG contributed his expertise in the literature on forgiveness and moral injury. NP prepared the initial draft of the manuscript, and all authors participated in revision and refinement of the final manuscript.

### Conflict of interest statement

The authors declare that the research was conducted in the absence of any commercial or financial relationships that could be construed as a potential conflict of interest.

## Footnotes

^1^We use the words “spirit” and “soul” in a general, inclusive sense without embracing a single definition. Here, spirituality might be thought of as a capacity for (or even a need to) find meaning and purpose in life. This meaning or purpose is often rooted in a sense of inter-connection with others or a belonging to a larger whole that is endowed with significance beyond one's own life. Spirituality is often, but not necessarily, tied to religious faith or beliefs. In the VA healthcare system (where we work), spiritual wellbeing is recognized as a core component of human health and wellness, and we seek to provide holistic “whole health” care that embraces the biological, psychological, social, and spiritual components of life and health (7). “Biopsychosocial-spiritual” care models (8) of this kind are advocated as a means to advance more integrated, person-centered healthcare (9).

^2^We use the term “soldier” in the generic, cross-cultural context to refer to anyone serving in a military.
